# Prevalence and Risk Factors Associated With Self-reported Psychological Distress Among Children and Adolescents During the COVID-19 Pandemic in China

**DOI:** 10.1001/jamanetworkopen.2020.35487

**Published:** 2021-01-26

**Authors:** Zuguo Qin, Lei Shi, Yaqing Xue, Huang Lin, Jinchan Zhang, Pengyan Liang, Zhiwei Lu, Mengxiong Wu, Yaguang Chen, Xiao Zheng, Yi Qian, Ping Ouyang, Ruibin Zhang, Xuefeng Yi, Chichen Zhang

**Affiliations:** 1 Health Publicity and Education Center of Guangdong Province, Guangzhou, China; 2 School of Health Services Management, Southern Medical University, Guangzhou, China; 3 School of Public Health, Southern Medical University, Guangzhou, China; 4 Department of Prevention and Health Care, Shantou Central Hospital/Affiliated Shantou Hospital of Sun Yat-sen University, Shantou, China; 5 Department of Medical Dispute, Maternal and Child Health Hospital, Heyuan, China; 6 Health Education Center of Maoming City, Maoming, China; 7 Department of Health Management, Nanfang Hospital, Southern Medical University, Guangzhou, China; 8 Department of Psychiatry, Zhujiang Hospital, Southern Medical University, Guangzhou, China; 9 Institute of Health Management, Southern Medical University Guangzhou, Guangzhou, China

## Abstract

**Importance:**

Schools have been suspended nationwide in 188 countries, and classes have shifted to home-based distance learning models to control the spread of the coronavirus disease 2019 (COVID-19) pandemic. Additional information is needed to determine mental health status among school-aged children and adolescents during this public health crisis and the risk factors associated with psychological distress during the pandemic.

**Objective:**

To assess self-reported psychological distress among school-aged children and adolescents associated with the COVID-19 pandemic.

**Design, Setting, and Participants:**

This cross-sectional study using data from a survey on the mental health of school-aged children and adolescents in Guangdong province, China, conducted by using a stratified cluster random sampling method between March 8 to 30, 2020. To estimate outcomes associated with location of districts, only data from students with internet protocol addresses and current addresses in Guangdong were included. Data were analyzed from April 5 to July 20, 2020.

**Exposure:**

Home-based distance learning during the COVID-19 pandemic.

**Main Outcome and Measures:**

The main outcome was self-reported psychological distress, measured using the total score on the 12-item General Health Questionnaire of 3 or greater. Multivariate logistic regression was used to analyze risk factors associated with mental health status. Odds ratios (ORs) were used to analyze the associations of factors with psychological distress.

**Results:**

Among 1 310 600 students who completed the survey, 1 199 320 students (mean [SD] age, 12.04 [3.01] years; 619 144 [51.6%] boys) were included in the final analysis. A total of 126 355 students (10.5%) self-reported psychological distress. Compared with students in primary school, high school students had increased risk of psychological distress (OR, 1.19 [95% CI, 1.15-1.23]). Compared with students who wore a face mask frequently, students who never wore a face mask had increased risk of psychological distress (OR, 2.59 [95% CI, 2.41-2.79]). Additionally, students who spent less than 0.5 hours exercising had increased odds of self-reported psychological distress compared with students who spent more than 1 hour exercising (OR, 1.64 [95% CI, 1.61-1.67]).

**Conclusions and Relevance:**

These findings suggest that the prevalence of self-reported psychological distress among students during the COVID-19 pandemic was relatively high. Frequency of wearing a face mask and time spent exercising were factors associated with mental health. Therefore, it may be necessary for governments, schools, and families to pay attention to the mental health of school-aged children and adolescents during the COVID-19 pandemic and take corresponding countermeasures to reduce the impact of the COVID-19 pandemic on students’ mental health.

## Key Points

**Question:**

What factors are associated with self-reported psychological distress among school-aged children and adolescents during the COVID-19 pandemic?

**Findings:**

In this cross-sectional study including 1 199 320 children and adolescents, the prevalence of self-reported psychological distress was 10.5%. Students who never wore a face mask were at higher risk for psychological distress compared with students who wore a face mask frequently, as were students who spent less than 0.5 hours exercising compared with students who spent more than 1 hour in exercising.

**Meaning:**

These findings suggest that the prevalence of self-reported psychological distress among school-aged children and adolescents during the COVID-19 pandemic was relatively high.

## Teaser

This cross-sectional study examines risk factors and prevalence of self-reported psychological distress among school-aged children and adolescents in China during the COVID-19 pandemic.

## Introduction

Since the first case of coronavirus disease 2019 (COVID-19) was reported in Wuhan, China,^1^ severe acute respiratory syndrome coronavirus 2 (SARS-CoV-2) was identified as the causative virus for the global pandemic.^2^ On March 11, 2020, the World Health Organization declared that the COVID-19 outbreak was a pandemic. Widespread outbreaks of COVID-19 were associated with psychological distress and symptoms of mental illness.^3^ Previous studies have observed moderate to severe psychological distress symptoms, such as depression and anxiety, among the populations in areas severely affected by the COVID-19 pandemic.^4,5^ Moreover, compounding factors, such as high mortality rate, shortage of medical protective materials, social isolation, and media information overload, could be associated with further adverse mental health outcomes during the pandemic.^6^ To date, the increasing attention toward on mental health status has shown that subsyndromal mental health problems are a common response to the COVID-19 pandemic.^3^ For example, a study by Wang et al^7^ found moderate to severe depressive symptoms in 16.5% of the general population in China, while moderate to severe anxiety symptoms were observed in 28.8% of the population and moderate to severe stress symptoms were observed in 8.1% of the population. Although many studies have focused on the general population,^7^ the mental health status of specific populations during the COVID-19 outbreak is attracting an increasing amount of attention. For example, Chen et al^8^ found that there was a negative association between internet-related behaviors and psychological distress among children in China during the COVID-19 pandemic. However, the power to determine the impacts of this unprecedented pandemic on the mental health status of adolescents is limited by the small sample size of previous studies. In addition, it is unclear what potential factors may be associated with risk of or protection from adverse outcomes in adolescents’ mental health status, and this lack of knowledge might impede the use of proper psychological interventions for adolescents.

Although children and adolescents are generally healthy and do not require much health care outside of regular checkups and immunizations, mental health care is critical for children and adolescents. Studies have consistently indicated that early identification and treatment is essential during this sensitive time in child development, or there may be adverse health and social outcomes.^9^ The COVID-19 pandemic may worsen existing mental health problems and be associated with more mental health problems among children and adolescents because of the unique combination of the public health crisis and social isolation.^10^ A 2020 study by Oosterhoff et al^11^ suggested that social distancing procedures involving minimizing social and physical contact between people during the COVID-19 pandemic was associated with significant mental health impacts. According to the United Nations Educational, Scientific and Cultural Organization,^12^ schools have been suspended nationwide in 188 countries, and classes have been shifted to home-based distance learning models to contain the spread of COVID-19. These unprecedented social distancing measures have affected more than 90% of enrolled learners, or approximately 1.5 billion young people worldwide. Although massive efforts are being made by schools and teachers at all levels to create online courses and deliver them through television broadcasts and the internet, prolonged school closure and home confinement during the COVID-19 pandemic may be associated with adverse mental health outcomes.^11^ Prolonged duration of shelter-in-place orders, fears of infection, inadequate information, lack of personal space at home, the efficacy of online learning, uncertainty about examinations and matriculation arrangements, family financial loss, and lack of in-person contact with classmates, friends, and teachers are examples of stressors that could have adverse and enduring outcomes among children and adolescents.^13^ A 2013 study found that posttraumatic stress disorder scores were 4-fold higher in children who had been quarantined than in those who were not quarantined.^14^ Moreover, the stress of routine daily life and the psychosocial stress associated with home confinement could further aggravate the detrimental impacts on mental health.^11^ Beyond mental health problems associated with home confinement, there is little knowledge about the mental health status and risk factors associated with the mental health of children and adolescents during the COVID-19 pandemic, which could impede educators, administrators, and policy makers from developing effective interventions to prevent adverse effects of home confinement.

To fill this gap, we examined data from a sample covering a wide age range from 1 Chinese province with more than 1 million school-aged children and adolescents. The aims of this study were to understand the self-reported psychological distress status among school-aged children and adolescents and to identify the risk and protective factors associated with self-reported psychological distress during the COVID-19 pandemic using a cross-sectional study with large sample.

## Methods

This study was approved by the ethics committee of Southern Medical University. A parent or guardian assented to student participation before they completed the survey. This study is reported following the American Association for Public Opinion Research (AAPOR) reporting guideline.

### Study Population

The target population comprised school-aged students in Guangdong province, China, which is 1 of the most developed provinces and has had the most cases of COVID-19 in China. A stratified cluster random sampling method was used to collect data between March 8 to 30, 2020. To build our sample, a 10% of primary and secondary schools from each city in Guangdong province were randomly selected sampling with equal probabilities method, then a cluster sampling method was used to extract students from these schools, and the probability of each student being selected was the same. Owing to the impact of the pandemic, the respondents in the target population completed the Chinese version of the electronic questionnaire through an online survey platform (SurveyStar; Changsha Ranxing Science and Technology). The questionnaires were anonymous to ensure the confidentiality and reliability of data. A total of 1 310 600 students completed the form, and 1 199 320 valid questionnaires were returned, resulting in a response rate of 91.5%. The survey link was sent to the cell phone of the child’s parent or guardian, and parents or guardians were asked to assent to their child’s participation in the survey before the child could participate.

### Assessment of the Mental Health Outcome

The mental health status of students was assessed using the Chinese version of 12-item General Health Questionnaire (GHQ-12).^15^ We chose the GHQ-12 because even if a diagnosis could not be determined from the GHQ-12, it could identify people who might be inclined to experience mental health problems (ie, depression, anxiety, or both). The GHQ-12 includes 6 negative questions and 6 positive questions, with responses ranging from not at all, no more than usual, rather more than usual, to much more than usual. The Chinese version of the GHQ-12 is a practical tool for assessing the mental health status of children and adolescents with good internal reliability and external validity^16^ and has been widely used in children and adolescents from different cultural backgrounds.^17,18^ Although several scoring formats have been proposed for the GHQ-12, in this study, we used a binary format (0 or 1). The total scores ranged from 0 to 12, with higher scores indicating worse psychological problems. GHQ-12 scores 3 or greater were determined to indicate self-reported psychological distress, but the score may trade off sensitivity and specificity for detecting individuals with mental distress.^19^ The Cronbach α coefficient for this scale in this present study was 0.75.

### Factors Associated With Mental Health During the COVID-19 Outbreak

We examined factors associated with mental health as 2 categories. The first category was characteristics not associated with the COVID-19 pandemic, ie, trait-like features unrelated to COVID-19. The second category was characteristics that may be influenced by the COVID-19 pandemic, ie, state-like features during the COVID-19 pandemic.

Characteristics not associated with the COVID-19 pandemic were sociodemographic characteristics that are typically found in school-aged youths, such as their sex, age, grade level, only child (yes or no), economic status (high, medium, or low, provided by the parent or guardian), location of residence (urban or suburb), and region of residence: Pearl River Delta, East Guangdong, West Guangdong, and North Guangdong. These 4 regions are divided according to the economic development level of Guangdong province.

Characteristics associated with the COVID-19 pandemic, such as weekly exercise time while confined at home (>1 hour, 0.5-1 hour, <0.5 hours), sources for COVID-19 information (1, 2-3, 4-5, >5), and frequency of face mask use (always, most of the time, sometimes, few, or never) on occasions when it would be recommended to wear a mask (eg, when shopping), were included. Regarding COVID-19 information sources, students were asked to make choices about possible sources for information about COVID-19, including through family members, friends, publicity in the community, broadcasts, television, internet, and or another means. Beyond knowledge transmission, students from undeveloped areas also might have limited resources to access online courses during the COVID-19 outbreak, which might further impact mental health status due to a sense of falling behind in learning. To test whether economic level was associated with mental status, we classified the living areas into 4 regions: Pearl River Delta, East Guangdong, West Guangdong, and North Guangdong. Finally, we coded each district with a different rank of risk for COVID-19 following the guidelines for partitioned and graded prevention and control of COVID-19 in Guangdong province.^20^

### Statistical Analysis

Categorical variables were presented as numbers and percentages and compared using a Pearson χ^2^ test or Fisher exact test between poor mental health (GHQ score ≥3) and good mental health (GHQ score <3) groups. *P* values for trends were calculated using the mental health variable as a binary categorical variable (1 = GHQ score ≥3, 0 = GHQ score <3) in the Pearson χ^2^ trend test and regression model trend test. *P* values were 2-sided, and statistical significance was set at *P* < .05.

To further examine potential factors associated with risk or protection for mental health status, a multivariate logistic regression analysis was used, and odds ratios (ORs) and 95% CIs were calculated. In this model, we included potential variables and set the significance level at *P* < .05. All analyses were performed with Stata statistical software version 15.0 (StataCorp) and R statistical software version 4.0.3 with *forestplot* package (R Project for Statistical Computing). To account for the clustering of participants, we weighted by geographic area of residence and stratified by sex. Data were analyzed from April 5 to July 20, 2020.

## Results

**Figure 1.**
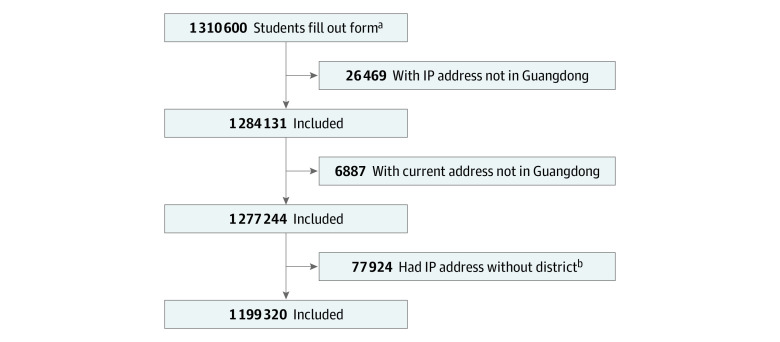
Flowchart of Participant Selection ^a^Owing to the coronavirus disease 2019 pandemic occurring during the winter vacation, some students went back to their hometown outside of Guangdong province. ^b^Some internet protocol (IP) addresses were in Guangdong province, but the city name was blocked, which would block the analysis of the geographic associations with psychological distress.

**Figure 2.**
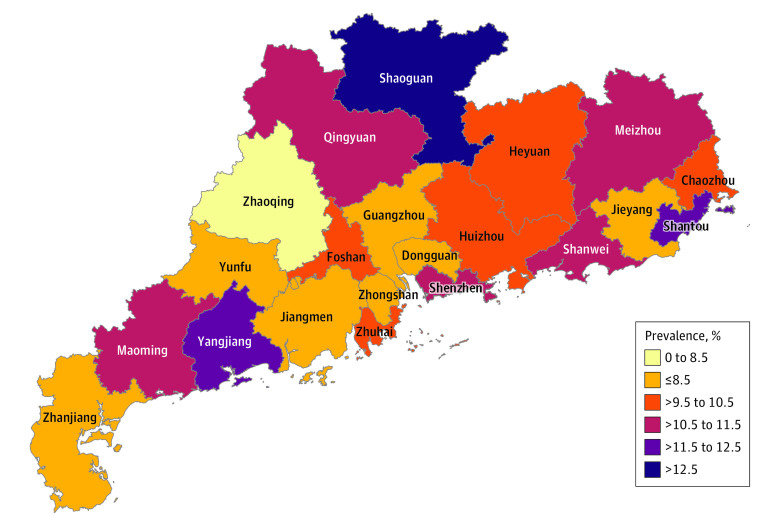
Prevalence of Psychological Distress Among School-Aged Children and Adolescents in Guangdong Province

**Figure 3.**
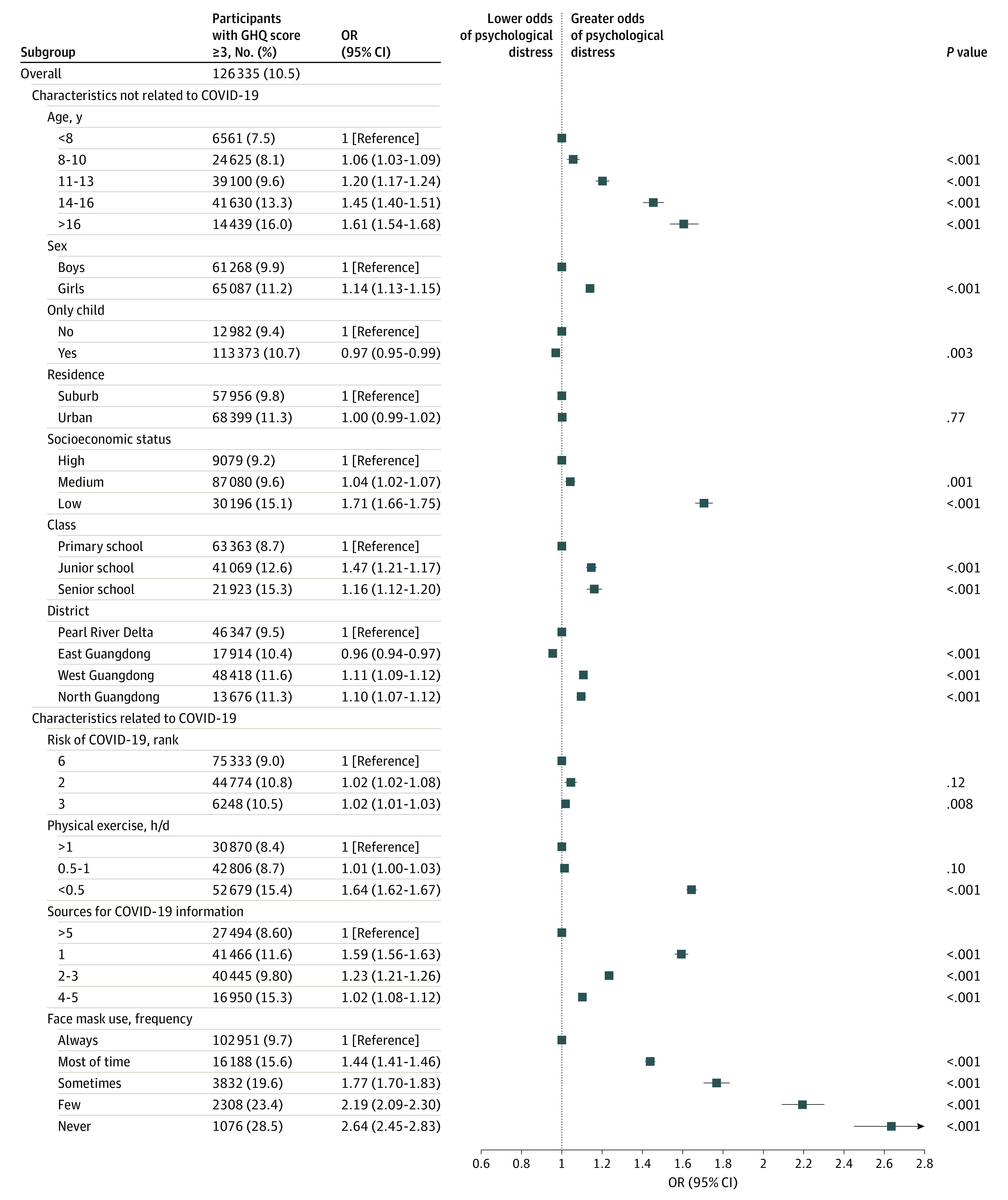
Factors Associated With Risk of or Protection From Self-reported Psychological Distress

A total of 1 310 600 students completed the questionnaire, and 1 199 320 respondents (mean [SD] age, 12.04 [3.01] years; 619 144 [51.6%] boys) respondents were included in the final analysis. A total of 126 355 students (10.5%) reported psychological distress, among whom 65087 (51.5%) were girls. A flowchart of the respondents can be found in Figure 1. Figure 2 shows the prevalence estimate for self-reported psychological distress among school-aged children and adolescents in 21 cities of Guangdong province. Pearson χ^2^ trend tests found that all examined characteristics, including those associated with the COVID-19 pandemic and those not associated with the pandemic, were associated with mental health status in a significant linear trend (Table 1). Based on these results, we conducted an additional analysis using multivariate logistic regression to identify risk factors associated with mental health status (Figure 3).

**Table 1.** Sociodemographic and COVID-19–Related Factors of Survey Respondents

Variables	GHQ ≥3 (n = 126 355 [10.5%])	GHQ <3 (n = 1 072 965 [89.4%])	*P* value for trend
Age, y			
<8	6561 (7.5)	80 627 (92.5)	<.001
8-10	24 625 (8.1)	278 580 (91.9)	
11-13	39 100 (9.6)	367 630 (90.4)	
14-16	41 630 (13.3)	270 415 (86.7)	
>16	14 439 (16.0)	75 713 (84.0)	
Sex			
Boys	61 268 (9.9)	557 876 (90.1)	<.001
Girls	65 087 (11.2)	515 089 (88.8)	
Only child			
Yes	12 982 (9.4)	124 472 (90.6)	<.001
No	113 373 (10.7)	948 493 (89.3)	
Economic status			
High	9079 (9.2)	89 914 (90.8)	<.001
Medium	87 080 (9.6)	817 927 (90.4)	
Low	30 196 (15.5)	165 124 (84.5)	
Grade level			
Primary school	63 363 (8.7)	666 643 (91.3)	<.001
Secondary school	41 069 (12.6)	284 795 (87.4)	
High school	21 923 (15.3)	121 527 (84.7)	
Residence			
Urban	57 956 (9.8)	535 920 (90.2)	<.001
Suburb	68 399 (11.3)	537 045 (88.7)	
District^a^			
Pearl River Delta	46 347 (9.5)	442 553 (90.5)	<.001
East Guangdong	17 914 (10.4)	154 894 (89.6)	
West Guangdong	48 418 (11.6)	368 453 (88.4)	
North Guangdong	13 676 (11.3)	107 065 (88.7)	
Risk rank of COVID-19^20^			
I	NA	NA	
II	6248 (9.0)	63 407 (91.0)	<.001
III	44 774 (10.8)	368 574 (89.2)	
VI	75 333 (10.5)	640 984 (89.5)	
Physical exercise, h/d			
>1	30 870 (8.4)	335 808 (91.6)	<.001
0.5-1	42 806 (8.7)	447 053 (91.3)	
<0.5	52 679 (15.4)	290 104 (84.6)	
Sources for COVID-19 information, No.			
1	16 950 (15.3)	93 767 (84.7)	<.001
2-3	41 466 (11.6)	314 771 (88.4)	
4-5	40 445 (9.8)	372 349 (90.2)	
>5	27 494 (8.6)	292 078 (91.4)	
Frequency of face mask using			
Always	102 951 (9.7)	959 427 (90.3)	<.001
Most of time	16 188 (15.6)	87 550 (84.4)	
Sometimes	3832 (19.6)	15 723 (80.4)	
Rarely	2308 (23.4)	7562 (76.6)	
Never	1076 (28.5)	2703 (71.5)	

Abbreviations: COVID-19, coronavirus disease 2019; NA, not applicable.

^a^ The categorical variables were tested by Fisher exact probability test.

### Characteristics Not Related to the COVID-19 Pandemic and Self-reported Psychological Distress

We found that sociodemographic factors, including age, sex, only child status, residence, economic status, school class, and areas where the students lived were significantly associated with the self-reported psychological distress before assigning weights. However, only child status and area of residence were not significantly associated with the self-reported psychological distress after assigning weights (Table 2). Older students were more likely to experience psychological distress, as high school students were more likely to report psychological distress compared with primary school students (OR, 1.19 [95% CI, 1.15-1.23]) (Figure 3), and those in their final year of high school had the highest prevalence of self-reported psychological distress (eFigure 2 in the Supplement). Furthermore, a positive linear association between age and the prevalence of self-reported psychological distress was found (eFigure 1 in the Supplement). Compared with students from the Pearl River Delta, students in West Guangdong (OR, 1.11 [95% CI, 1.09-1.12]) and North Guangdong (OR, 1.10 [95% CI, 1.07-1.12]) had higher odds of psychological distress. In line with economic development levels, compared with students who had high economic status, students with medium economic status (OR, 1.04 [95% CI, 1.02-1.07]) and low economic status (OR, 1.71 [95% CI, 1.66-1.75]) had higher odds of psychological distress (Figure 3).

**Table 2.** Multivariate Logistic Regression Analysis of Characteristics Associatated With Psychological Distress Among School-Aged Children and Adolescents During the COVID-19 Pandemic in Guangdong Province, China

Variables	Unadjusted OR (95% CI)^a^	*P* value	Adjusted OR (95% CI)	*P* value
Characteristics not related to COVID-19				
Age	1.16 (1.15-1.17)	<.001	1.16 (1.10-1.23)	.008
Sex	1.12 (1.10-1.13)	<.001	1.12 (1.10-1.14)	.002
Only child	0.97 (0.95-0.99)	.002	0.96 (0.79-1.15)	.41
Economic status	1.43 (1.42-1.45)	<.001	1.44 (1.27-1.64)	.006
Grade	1.10 (1.08-1.11)	<.001	1.10 (1.06-1.14)	.008
Residence	0.96 (0.95-0.97)	<.001	0.97 (0.90-1.04)	.18
District	1.05 (1.04-1.06)	<.001	1.05 (1.04-1.06)	.004
Characteristics related to COVID-19				
Risk rank of COVID-19^20^	1.02 (1.01-1.03)	<.001	1.02 (1.00-1.04)	.03
Physical exercise	1.32 (1.31-1.33)	<.001	1.33 (1.21-1.46)	.006
Sources for COVID-19 information	1.01 (1.01-1.02)	<.001	1.01 (1.01-1.02)	.008
Frequency of face mask use	1.37 (1.36-1.39)	<.001	1.39 (1.18-1.64)	.01

Abbreviations: COVID-19, coronavirus disease 2019; OR, odds ratio.

^a^ AUC = 0.624 and Pseudo *R*^2^ = 0.029 in the logistic model.

### Characteristics Related to the COVID-19 Pandemic and Self-reported Psychological Distress

We observed that the factors related to COVID-19 were significantly associated with self-reported psychological distress (Table 2). Specifically, compared with students from areas coded as low risk for COVID-19 (rank VI), students from an area coded as high risk (rank II) had higher odds of psychological distress (OR, 1.02 [95% CI, 1.01-1.03) (logistic model: AUC = 0.641; Pseudo *R*^2^ = 0.037). Additionally, knowledge about sources for information on COVID-19 was associated with mental health status. Compared with students who knew more than 5 ways of finding knowledge about COVID-19, students who knew 1 way had higher odds of psychological distress (OR, 1.59 [95% CI, 1.56-1.63]), as did students who knew 2 to 3 ways (OR, 1.23 [95% CI, 1.21-1.26]), and those who knew 4 to 5 ways (OR, 1.10 [95% CI, 1.08-1.12]). Interestingly, we found that frequency of wearing a face mask and time spent exercising were associated with protection from adverse mental health. Compared with students who always wore face masks, students who rarely wore face masks had significant higher odds of self-reported psychological distress (OR, 2.59 [95% CI, 2.41-2.79]). Compared with students who spent more than 1 hour exercising. students who spent less than 0.5 hours exercising also had higher odds of self-reported psychological distress (OR, 1.64 [95% CI, 1.62-1.67]) (Figure 3).

## Discussion

This cross-sectional study examined prevalence and risk factors associated with self-reported psychological distress among primary and secondary students during the COVID-19 pandemic based on a large sample from Guangdong province, China. To our knowledge, few studies have explored factors associated with mental health status among children and adolescents during the COVID-19 pandemic using a sample of more than 1 million participants. This study found an overall prevalence of self-reported psychological distress of 10.5%. Moreover, our findings indicated that the frequency of wearing a face mask and time spent exercising had protective associations with mental health.

The prevalence of self-reported psychological distress among school-aged children and adolescents during the COVID-19 pandemic was higher than the prevalence of mental health problems among children and adolescents in Guangzhou reported in a 2017 study,^21^ before the COVID-19 pandemic, but lower than psychological distress reported in the general population of Guangzhou during the COVID-19 pandemic.^22^ This could be the result of numerous factors. First, the COVID-19 pandemic forced school-aged children and adolescents to stay at home, and they could not play with their friends, which may have increased the incidence of perceived loneliness and exacerbated its affects. During the outbreak of COVID-19, schools were suspended, and all students were instructed to study at home through online videos. In such cases, outdoor activities and social interaction were largely reduced, which could increase depressive symptoms.^23^ A study by Oosterhoff et al^11^ suggested that the government’s social distancing policy during the COVID-19 outbreak was associated with psychological distress among adolescents. Second, exposure to family members continually discussing the numbers of COVID-19 cases and deaths, may have led to increased anxiety among children and adolescents. In addition, a study by Campbell^24^ suggested that actions attempting to reduce the spread of COVID-19, such as social distancing, sheltering in place, restricted travel, and closures of key community resources, were likely to dramatically increase the risk of family violence.^24^ Furthermore, students who take online course by smartphone or computer during the school suspension, especially if parents and teachers do not monitor smartphone and social media use, might engage in problematic application or social media use. A study by Chen et al^8^ found greater associations between problematic smartphone-application use, problematic social media use, and psychological distress during the COVID-19 pandemic compared with findings among adolescents who participated in online learning at home prior to the pandemic.

Previous studies have shown that the prevalence of psychological distress is variable.^25,26^ It is challenging to compare the prevalence of psychological distress among school-aged children and adolescents from different studies^11^ owing to inconsistencies in the culture context, samples, methods or scales used, or use of same scales but different cutoff values to measure psychological distress. Our results from a sample of more than 1 million participants from 1 province suggest the general mental health status of primary and secondary students showed consistency with other studies set in China.^21,22^ This would further support the need for psychological intervention strategies for students during COVID-19 that go beyond the usual care practiced before the COVID-19 pandemic.

Regarding the sociodemographic factors not related to the COVID-19 pandemic, we found that older students were more likely to experience psychological distress, and those in their final year of high school had the highest prevalence of self-reported psychological distress. One possible reason is that in the context of Chinese culture, the college entrance examination is not only important for the students themselves but also is a pivotal event for the family.^27^ High school students, especially those in their final year of high school, may be not only worried about their review plans being disrupted during the COVID-19 pandemic, but also may experience pressure from their parents, relatives, and friends, which may further exacerbate any psychological distress. In addition, students from less developed areas had a higher incidence of psychological distress. In line with these results, students with a low family economic status may face more serious psychological problems during the COVID-19 pandemic. This phenomenon may be attributed to the fact that families with poor economic conditions may be facing even more financial difficulty during the COVID-19 pandemic and may be restricted in living standards and diet or nutrition, so that negative emotions from the parents may be spread to children and adolescents, which may in turn increase psychological burden. Studies have demonstrated that individuals from families with low economic status are more likely to experience psychological distress.^28,29^ In addition, students who come from less developed areas and have a poor economic status might have limited resources to access the online courses, which might further impact the mental health status owing to a sense of falling behind in learning.

Regarding the factors associated with the COVID-19 pandemic, we found higher odds of psychological distress among school-aged children and adolescents in a high-risk area (ie, rank II) compared with those in a low-risk areas (ie, rank VI). In line with these findings, the results from a study by Xie et al^23^ that recruited children from Hubei province showed that the children from Wuhan, China, the city where COVID-19 was first recorded and with the most cases of COVID-19 in Hubei province, had significantly higher depression status scores than children in Huangshi, another city in Hubei with fewer COVID-19 cases. Some potential reasons for this difference include that school-aged children and adolescents in high-risk areas may worry about their families, relatives, and friends being infected, in addition to worrying about themselves getting infected. Meanwhile, school-aged children and adolescents in high-risk areas also may receive negative information about the number of people infected in the area every day, which may further increase their psychological distress.

However, other COVID-19–related factors, such as knowledge about COVID-19, face mask–wearing frequency, and time spent exercising were associated with protection from psychological distress. Students who had more sources for information on COVID-19 were less likely to experience psychological distress. Often it is the unknown that is frightening, so students who have multiple sources for COVID-19–related information (eg, how to prevent COVID-19, transmission mechanisms of SARS-CoV-2) that they can understand may reduce psychological distress. Interestingly, we found that the frequency of wearing a face mask and time spent exercising had a protective association with mental health. Moreover, the association of overall frequency of mask use with self-reported psychological distress may reflect whether students are leaving their home frequently. It might also reflect the preference of the student or of their parents and the cost of masks at that time. Wearing a face mask is one of the main ways individuals can prevent infection.^30,31^ Students who wear face masks frequently might feel less likely to contract COVID-19, which could further reduce worry and anxiety levels and promote mental well-being. Therefore, wearing a mask may be more conducive to mental health. Of course, there are various potential reasons for the associations of mental health with COVID-19 knowledge and face mask use, some of which imply no causal effect. For example, lower COVID-19 knowledge could reflect lower intelligence of the student or household characteristics that may in turn be associated with poor mental health. At the same time, students with preexisting mental health problems may pay less attention to COVID-19 and therefore be less likely to learn about its transmission or be concerned about mask use. In addition, children and adolescents who spent less time exercising were more likely to experience psychological distress, which is consistent with a previous large sample study in the United States between 2011 and 2015.^32^ During home confinement, exercise could improve mood and self-confidence of children and adolescents, as exercises has been shown to have beneficial associations with the prevention and treatment of psychiatric diseases and could promote mental well-being.^33^

### Limitations

This study has some limitations. The cross-sectional nature of this study limits the ability to establish the direction of causality for the association between the risk factors and mental health. In other words, a more distressed person might be less inclined to take self-protective steps. It is necessary to perform further longitudinal research to provide additional explanations for these associations. Additionally, we used an internet survey because of the COVID-19 pandemic, but the reliability of the survey results is greatly affected by the participants. However, our study covered all students from primary school to senior high school in Guangdong province, and the overall sample size was large and representative, which can help extend our results to reflect the mental health of school-aged children and adolescents during the COVID-19 pandemic.

## Conclusions

In this cross-sectional study in a large sample from Guangdong province, the prevalence of psychological distress among school-aged children and adolescents during the COVID-19 pandemic was relatively high. Age, family economic status, and risk level for a COVID-19 outbreak were risk factors associated mental well-being. Additionally, the frequency of wearing a face mask and time spent exercising had protective associations for mental health. Based on these findings, it is necessary for governments, schools, and families to pay attention to the mental health of school-aged children and adolescents during the COVID-19 pandemic and take appropriate countermeasures to reduce the impact of the COVID-19 pandemic on mental health for children and adolescents.

## Notes

Supplement. **eFigure 1.** Prevalence and Risk Factors Associated With Psychological Distress Among School-Aged Students During the COVID-19 Pandemic **eFigure 2.** Association of Grade Level With Risk of Psychological Distress **eTable.** 12-Item General Health Questionnaire
