# Supplementary material for: Prevalence and Risk Factors Associated With Self-reported Psychological Distress Among Children and Adolescents During the COVID-19 Pandemic in China
Source: JAMA Netw Open. 2021 Jan 26;4(1):e2035487. doi: 10.1001/jamanetworkopen.2020.35487 (PMC7838937; doi:10.1001/jamanetworkopen.2020.35487)
Supplement: Supplement. — eFigure 1. Prevalence and Risk Factors Associated With Psychological Distress Among School-Aged Students During the COVID-19 Pandemic eFigure 2. Association of Grade Level With Risk of Psychological Distress eTable. 12-Item General Health Questionnaire [file jamanetwopen-e2035487-s001.pdf]

Supplemental Online Content

Qin Z, Shi L, Xue Y, et al. Prevalence and risk factors associated with self-reported psychological distress among children and adolescents during the COVID-19 pandemic in China. *JAMA Netw Open*. 2021;4(1):e2035487.
doi:10.1001/jamanetworkopen.2020.35487

eFigure 1. Prevalence and Risk Factors Associated With Psychological Distress Among School-Aged Students During the COVID-19 Pandemic

eFigure 2. Association of Grade Level With Risk of Psychological Distress

eTable. 12-Item General Health Questionnaire

This supplemental material has been provided by the authors to give readers additional information about their work.

© 2021 Qin Z et al. *JAMA Network Open.*

eFigure 1. Prevalence and Risk Factors Associated With Psychological Distress Among School-Aged Students During the COVID-19 Pandemic

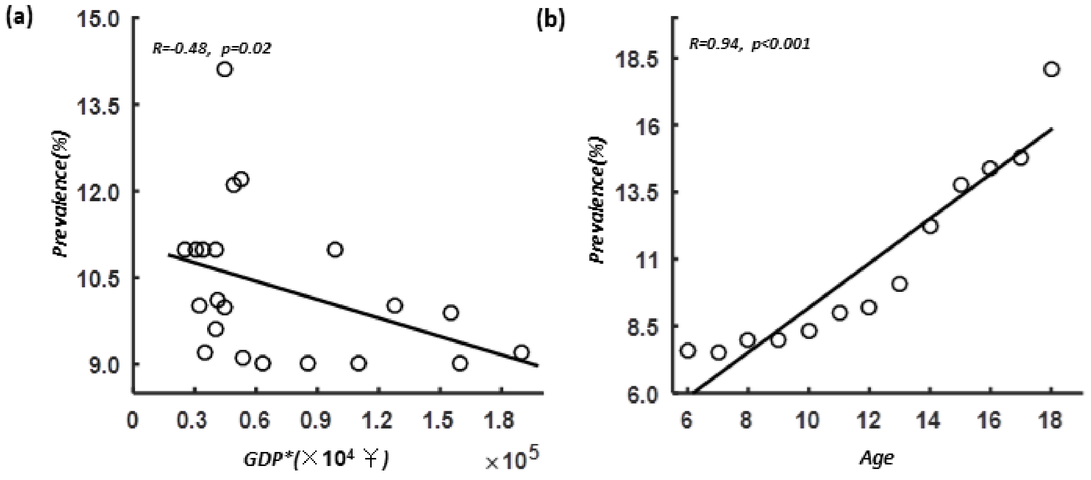

Fig. S1 The association between the GDP and the prevalence of psychological distress (a) and the age and prevalence (b). Note, *, the data released 2018.

© 2021 Qin Z et al. *JAMA Network Open.*

eFigure 2. Association of Grade Level With Risk of Psychological Distress

Subgroup	No. of GHQ≥3 (%)	Odd ratio	Low CI	High CI	P Value
Primary school					
Year 1 class	9361 (7.9)	reference			
Year 2 class	8510 (7.9)	0.998	0.968	1.029	0.9
Year 3 class	8694 (8.4)	1.074	1.042	1.107	<0.001
Year 4 class	11673 (8.8)	1.12	1.089	1.153	<0.001
Year 5 class	12511 (9.1)	1.166	1.134	1.199	<0.001
Year 6 class	12614 (9.7)	1.246	1.212	1.282	<0.001
Junior high School					
First year	13612 (11.2)	1.468	1.428	1.509	<0.001
Second year	13191 (12.4)	1.647	1.602	1.694	<0.001
Third year	14266 (14.6)	1.987	1.934	2.043	<0.001
Senior High school					
First year	8768 (14.4)	1.96	1.901	2.022	<0.001
Second year	6897 (13.5)	1.82	1.761	1.881	<0.001
Third year	6258 (19.8)	2.879	2.781	2.981	<0.001

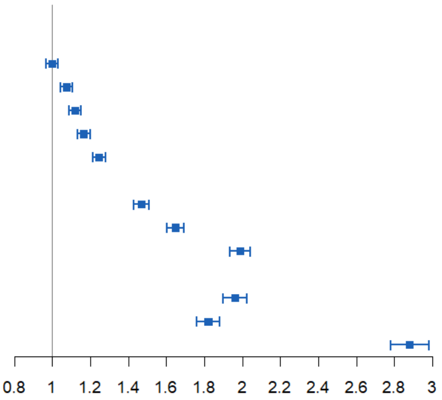

Fig. S2 Relative to the first year of primary school, individuals who at high class had the high incident rate experiencing psychological distress.

© 2021 Qin Z et al. *JAMA Network Open.*

eTable. 12-Item General Health Questionnaire

Items	Not at al	No more than usual	Rather more than usual	Much more than usual
1.Can you concentrate on what you are doing?	4	3	2	1
2.Do you have insomnia due to excessive worry?	1	2	3	4
3.Do you think you are useful?	4	3	2	1
4.Do you feel like you're decisive?	4	3	2	1
5.Always in a state of tension?	1	2	3	4
6.Do you think you can't solve the problem?	4	3	2	1
7.Can you enjoy your daily activities?	4	3	2	1
8.Can you face the problems you face?	4	3	2	1
9.Do you feel pain or worry?	1	2	3	4
10.Have you lost confidence?	1	2	3	4
11.Do you think you are worthless?	1	2	3	4
12.Do you think everything is going well?	4	3	2	1

© 2021 Qin Z et al. *JAMA Network Open.*
